# See more, detect more: investigating colonic inspection techniques and polyp detection using machine learning

**DOI:** 10.1007/s00464-026-13204-7

**Published:** 2026-08-04

**Authors:** Isra K. Elsaadany, Sanaz Motamedi, Hang-Ling Wu, Scarlett R. Miller

**Affiliations:** 1 Department of Industrial and Manufacturing Engineering, Penn State, 216 Leonhard Building, University Park, PA 16802 USA; 2 Department of Mechanical and Nuclear Engineering, Penn State, University Park, PA 16802 USA

**Keywords:** Vision transformer machine learning, Colonoscopy withdrawal, Colonoscopy inspection techniques, Colonoscopy simulation-based training, Computer vision classification model, Polyp detection colonoscopy

## Abstract

**Background:**

Colorectal cancer (CRC) is a leading cause of cancer mortality despite widespread colonoscopy screening. Colonoscopy effectiveness, which can reduce CRC risk by 90%, depends largely on adenoma detection rate (ADR), a metric strongly influenced by mucosal inspection quality during withdrawal. However, ADR miss rates remain as high as 26% due to incomplete inspection. Although simulation-based training (SBT) improves procedural skills, physical simulators lack objective assessment and feedback on inspection quality. This study aimed to develop an automated machine-learning model (ML) to objectively assess colonoscopy inspection performance and identify feedback to improve ADR.

**Methods:**

A Vision Transformer (ViT)-based ML model was developed to automatically recognize three inspection techniques during SBT required for effective inspection: clear lumen view, lateral wall, and obscured (fold) inspection. Model performance was evaluated using accuracy, precision, recall, and F1 score. The validated model was applied to videos from experts (*n* = 5), intermediate (*n* = 5), and novice residents (*n* = 7) performing withdrawal on a physical simulator embedded with silicone polyps. The model quantified time spent in each technique and surrounding polyp detection. Polyp detection rate (PDR) was quantified based on number of polyps detected.

**Results:**

The model achieved high accuracy, precision, recall, and F1 score. Greater inspection time in obscured regions was significantly associated with higher PDR (*r* = 0.50, *p* = 0.041). Compared with novices, experts spent more time inspecting with a clear lumen view (*p* = 0.039) and deeper obscured views (*p* = 0.017), demonstrated higher PDR, and devoted more time to obscured regions before or after polyp detection.

**Conclusion:**

The ViT ML model accurately recognizes key inspection techniques and objectively differentiates inspection performance across expertise levels using time-based metrics and PDR. This enables objective assessment and targeted feedback—such as highlighting uninspected, obscured regions within physical SBT, with the potential to improve inspection performance and ADR.

Colorectal cancer (CRC) is the third leading cause of cancer-related death in the United States, with around 53,000 deaths in 2025 [1]. Early detection through preventive screening can drastically improve outcomes for CRC [2] and reduce CRC mortality rates [3]. Global predictions have also indicated that the number of deaths from CRC will grow by 60–70% until 2035 [4] with the mean age of incidence trending downwards [5]. In response to the growing burden and the benefits of earlier detection [3], the American Cancer Society (ACS) has decreased the recommended age for colon cancer screening from 50 to 45 years old in 2018 [6]. This shift, paired with an increased public awareness of CRC, has led to a high volume of procedures- over 15 million colonoscopies are performed annually for CRC screening to detect adenomas in the United States [7]. Because adenomas are precursors to CRC, their timely identification and removal interrupt the adenoma–carcinoma sequence [8]. Colonoscopy remains the gold standard for clinical diagnosis [9], reducing CRC risk by 90% [10] and mortality rates [3]. However, the effectiveness of a colonoscopy depends not only on whether the procedure is performed, but also on the quality of the examination [11]. Prior studies have reported significant variability in colonoscopy quality in routine practice, where incomplete examinations and missed lesions may contribute to post-colonoscopy colorectal cancer (PCCRC) [11, 12]. This highlights the need to improve the consistency and quality of colonoscopy screening.

The effectiveness of colonoscopy screening in detecting adenomas depends on the endoscopist’s ability to advance the colonoscope to the cecum and carefully withdraw it to inspect the entire mucosa for pre-cancerous adenomas [13]. Specifically, adenoma detection rate (ADR) is a key quality indicator for colonoscopy and a 1% increase in ADR has been linked to a 3% reduction in CRC [14], making ADR a direct measure of mucosal inspection quality [15]. Achieving an ADR of 25% requires extensive procedural experience—approximately 450 colonoscopies to reach an ADR of 25% [16] far exceeding the minimum of 50 procedures during general surgery residency [17], thereby increasing the risk of missed adenomas. Maximizing ADR also remains challenging, particularly for polyps hidden behind folds, and small polyps (1–5 mm), which have miss rates up to 26% [18]. These misses are attributed to incomplete mucosal inspection, especially of folds and flexures [19], and to the endoscopist’s level of experience [20], with more experience linked to higher detection rates [21]. Some key expert techniques for improving mucosal inspection and ADR include: (1) clear lumen view, with the distant lumen fully visible and the endoscope centered [22, 23]; (2) wall view, inspecting lateral colon walls with partial lumen visibility; and (3) obscured view, examining deeper or difficult regions such as folds and bends where the lumen is not visible [24–27]. Meticulous inspection behind folds and flexures improves ADR [28]. Effective colonoscopy requires a combination of these three complementary inspection techniques to achieve thorough mucosal examination of the mucosa for polyps [24, 25]. Specifically, more experienced surgeons employ more comprehensive mucosal inspection techniques and varied endoscope movements [29]. Prior studies also emphasize achieving clear luminal view as a core skill [30] and that proper positioning of the colonoscope tip is essential for optimal visualization during withdrawal [31], as effective inspection requires actively redirecting the endoscope from the lumen center toward the lateral walls [32]. In addition, failure to inspect behind folds can negatively impact ADR [33, 34]. Collectively, these findings highlight the need for targeted training that develops comprehensive mucosal inspection skills.

Simulation-based training (SBT) has been used to improve colonoscopy performance by reducing procedure time and error rates, increasing completion rates [35], and may enhance ADR [36]. While virtual reality (VR) simulators offer objective assessment and feedback [37], with feedback shown to improve ADR [38], they lack realistic haptics [39]. On the other hand, physical simulators provide superior haptic realism but rely on subjective expert feedback and lack automated performance feedback and assessment [40]. Emerging technologies, particularly machine learning (ML) models, offer avenues to automate and personalize assessment and feedback delivery. Over the past decade, ML has become central to surgical skill assessment [41]. In particular, Vision Transformer (ViT) models have demonstrated strong performance in medical image classification by capturing complex spatial relationships within visual data [42]. Building on this, the goal of this exploratory feasibility study was to improve colonoscopy training by providing automated objective assessment of inspection performance and targeted feedback within physical SBT, to improve mucosal inspection quality and ADR. To achieve this, we leveraged ML by training and validating a ViT model to automatically recognize the three key inspection techniques during colonoscopy withdrawal.

## Research questions (RQ)

The study was conducted in two phases. Phase 1 involved training and validating a ViT-based Computer Vision Classification Model (CVCM) to automatically recognize the three key inspection techniques during colonoscopy withdrawal and quantify inspection time in each technique. The outputs from Phase 1 were then applied in Phase 2 to address the following research questions (RQs):

### RQ1: Does the time spent in each inspection technique during withdrawal relate to polyp detection rate (PDR), and can the proportion of inspection time across techniques predict PDR?

The first research question examined whether inspection time in each technique is associated with PDR, and whether the proportion of time spent in each technique can predict PDR. The three inspection techniques were: (1) inspecting the mucosa with a clear lumen view, with the distant lumen fully visible and the endoscope centered; (2) wall view, inspecting lateral colon walls; and (3) obscured view, examining deeper or difficult regions such as folds and bends. It was hypothesized that longer inspection times would reflect more thorough mucosal visualization and therefore be linked to higher PDR, as complete inspection requires use of all three techniques [24]. Specifically, increased time in Technique 3 was expected to be most predictive of higher PDR, given evidence that meticulous inspection behind folds and flexures improves ADR [28].

### RQ2: Does PDR and inspection time across the three techniques differ between experts, intermediates, and novice residents during colonoscopy withdrawal?

The second research question examined whether PDR and inspection time across the three techniques differ by expertise level. It was hypothesized that experts would demonstrate higher PDR than novices, consistent with evidence linking higher detection rates to experience level [21]. It was further hypothesized that experts would have higher inspection times in each technique compared to residents, since prior findings show that experts employ more comprehensive mucosal inspection techniques and varied endoscope movements during training [29].

### RQ3: Do experts, intermediates, and novices differ in the time spent inspecting in each technique before and after polyp detection during withdrawal?

The third research question examined whether experts compared to residents differ in inspection time across the three techniques before and after polyp detection. It was hypothesized that experts would devote more time to Technique 1 and Technique 3 both before and after detection. This was based on evidence that complete mucosal inspection requires all three techniques and that lower miss rates are associated with careful examination behind folds and flexures [28]. Accordingly, we anticipated that experts, who typically demonstrate lower miss rates, would spend more time inspecting deeper regions to avoid missing additional polyps hidden nearby.

## Materials and methods

**Fig. 1:**
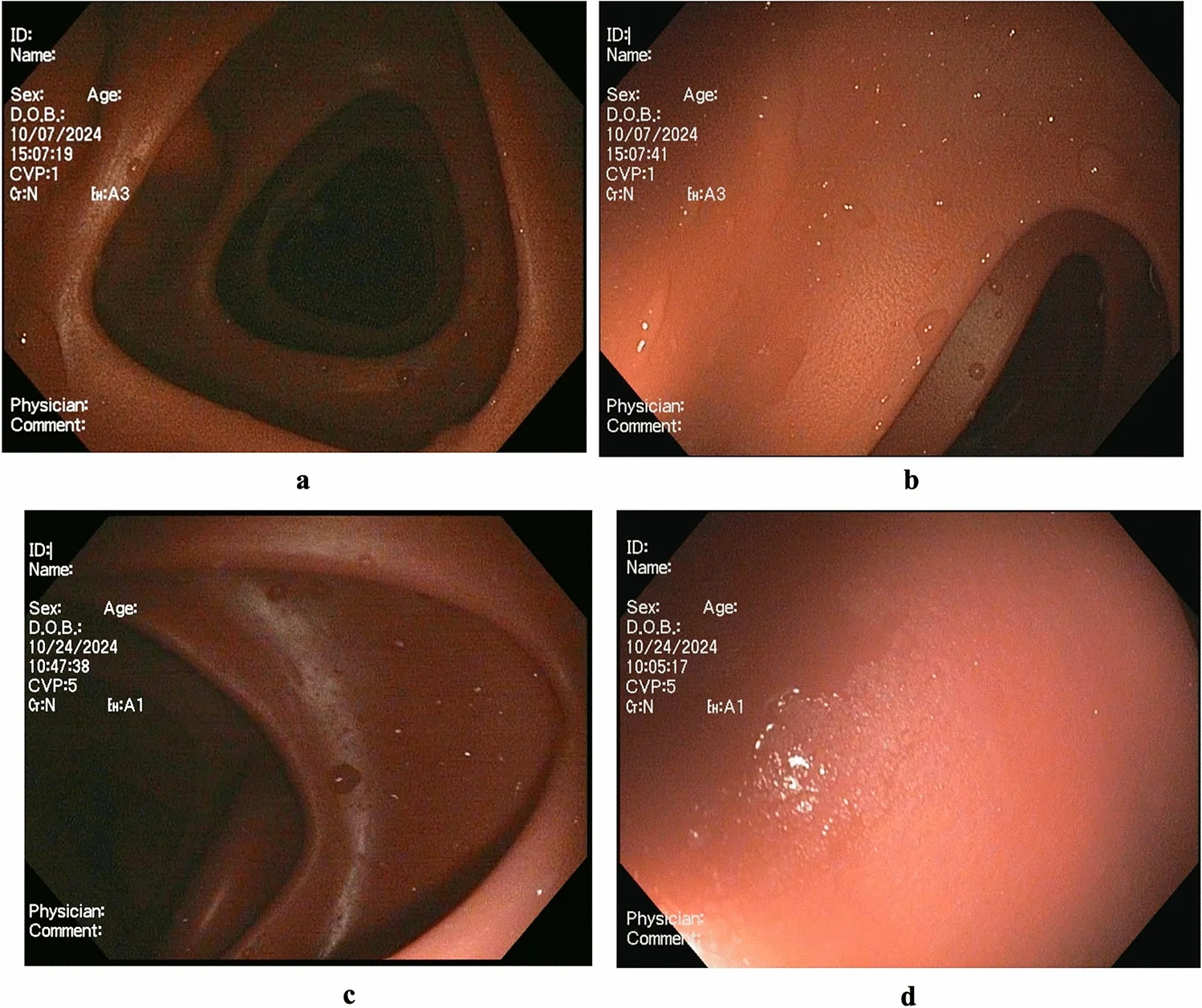
The three inspection techniques: Technique 1: clear lumen view with endoscope centered and distant colon is visible **a**, Technique 2: lateral wall inspection when lumen is partially visible **b**, **c**, and Technique 3: inspection of deeper, more obscured regions when lumen was not visible **d**

The aim of this study was to improve colonoscopy training by providing automated assessment of inspection performance and identifying targeted feedback that may enhance mucosal inspection skills and ADR. Phase 1 involved training and validating a ViT-based model to automatically classify the three key inspection techniques during withdrawal and quantify time in each technique. Phase 2 used the resulting CVCM-derived time metrics to answer our RQs. The three inspection techniques are illustrated in Fig. 1.

### Phase 1: training and validating a ViT CVCM

**Fig. 2:**
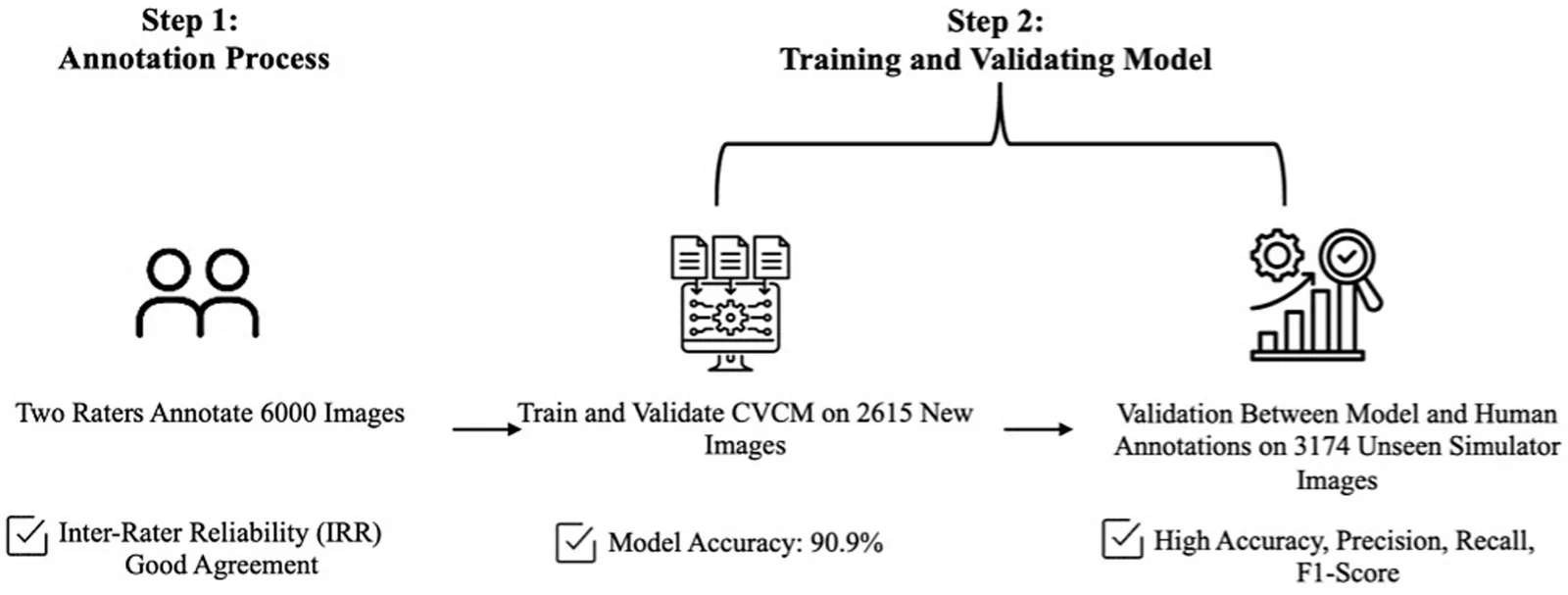
Annotation, training, and validation pipeline for the ViT classification model, including inter-rater reliability assessment and model–human validation on unseen simulator images

To develop the CVCM, colonoscopy simulator withdrawal videos were collected. Details on participants and the data collection procedure are provided in Sect. "Participants" and "Procedure". As shown in Fig. 2, two raters first annotated 6,000 simulator images to establish good inter-rater reliability (IRR), after which 2,615 images were used for training and calculating model accuracy. The model was then validated on 3,174 unseen simulator images using standard performance metrics. The following sections describe the annotation, training, and validation procedures.

#### Step 1: annotation process

Ground-truth labels were first established through dual, independent annotation. Two raters reviewed frames from a randomly selected withdrawal video to align on the criteria for each inspection technique, then independently labeled two additional videos sampled at the frame rate of the video (20 fps) (6,000 images). Inter-rater reliability was assessed using Cohen’s κ, resulting in good agreement (κ = 0.76, 95% CI [0.70, 0.82], *p* < 0.001) [43]. Discrepancies were resolved collaboratively to ensure consistent labeling before training the CVCM.

#### Step 2: training and validating the ViT CVCM

**Fig. 3:**
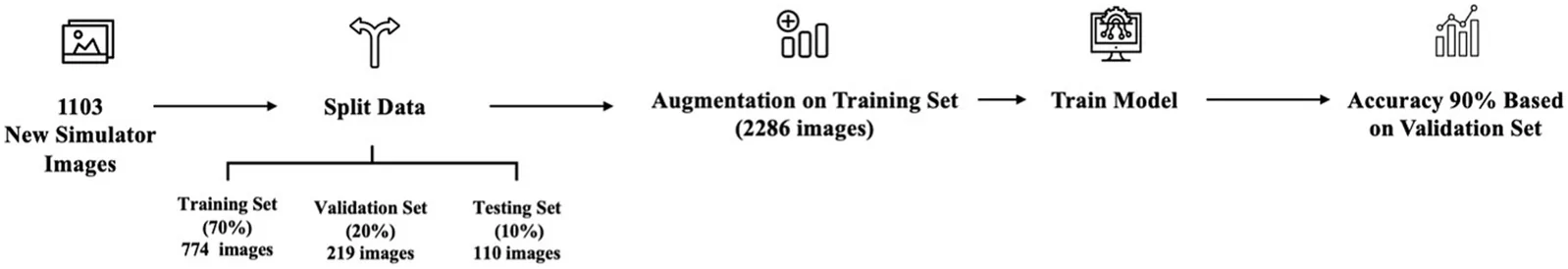
Overview of the Vision Transformer (ViT) model development pipeline. A total of 1,103 simulator images were split into training (70%), validation (20%), and testing (10%) sets. The training set was augmented to 2,286 images prior to model training, and model performance was evaluated on the validation set, achieving 90% accuracy

After achieving consistency in labeling, the ViT CVCM was trained using Roboflow, a platform for labeling and developing pre-built computer vision models with relative ease [44]. A new randomly selected simulator withdrawal video was processed at 20 fps, yielding 1,103 frames, which were manually labeled by a previous rater. The dataset was split into training (70%), validation (20%), and testing (10%) sets [45] with both validation and test sets treated as unseen data. To improve generalization, Roboflow’s augmentation pipeline applied random brightness adjustments (0–18%), generating three variants per training image and expanding the dataset to 2,615 images [46]. The model was trained with a 50% confidence threshold, consistent with prior work [47] and achieved 90.9% accuracy based on the validation data set. Steps are summarized in Fig. 3.

#### Result of the trained CVCM

The accuracy of the CVCM was found to be 90%. Precision, Recall, and F1 score for each inspection technique was calculated, with results summarized in Table 1. Overall, the model demonstrated strong precision and recall across all inspection techniques, with well-balanced performance reflected by consistently high F1-scores.

**Table 1:** Precision, recall and F1 score model performance results for each inspection technique

Inspection technique	Precision	Recall	F1 score
Technique 1	97.6%	80.8%	88.4%
Technique 2	85.8%	96.9%	91.0%
Technique 3	84.9%	100%	91.9%

#### Step 4: extracting total inspection time by technique

After validation, the CVCM was applied to all withdrawal videos. Frames were extracted at 20 fps using Python, labeled by the model, and technique durations calculated by dividing frame counts by the video frame rate.

#### Reproducibility and model implementation

A single-classification Vision Transformer (ViT) model was trained using the Roboflow platform. Training was conducted for approximately 17 epochs. The trained model weights and implementation details can be made available upon request to support reproducibility.

### Phase 2: analysis of inspection techniques during colonoscopy withdrawal

Once the model was validated, Phase 2 used the CVCM-derived time metrics to examine: (1) associations between inspection time per technique and PDR, (2) whether time proportions predicted PDR, and (3) differences in overall and pre/post polyp inspection time between experts and residents.

#### Outcome measures

The following metrics were used to answer our research questions:

Polyp Detection Rate (PDR): Defined as the number of detected polyps divided by the total inserted in the colon (15), see 3.2.2. During withdrawal, participants kept the endoscope near each detected polyp to ensure visibility. PDR was calculated by reviewing each video frame and counting distinct polyps appearing on the screen.

Withdrawal time: Defined as the duration from inspection at the cecum to endoscope removal.

Total Inspection Time by Technique: Defined as total time inspecting in each technique during withdrawal.

Proportion of Inspection Time by Technique: Defined as the total inspection time by technique divided by withdrawal time.

Pre- and Post-Polyp Inspection Time by Technique: This metric captured time in Technique 1–3 within a defined window before and after each polyp detection. To establish this window, each participant’s first polyp detected was excluded to minimize acclimation effects. To ensure inspection time across techniques was not affected by the appearance of another, any pair of polyps occurring within 5 s of each other was removed, chosen arbitrarily due to the lack of prior knowledge on typical detection intervals. This resulted in 104 polyps for analysis. Next, the minimum interval between consecutive polyp detections across all participants was 6 s, which was selected as the analysis window. All frames within 6 s before and after each polyp were manually labeled, and time in each technique was calculated by dividing the number of labeled frames by the video frame rate (20fps).

#### Apparatus

**Fig. 4:**
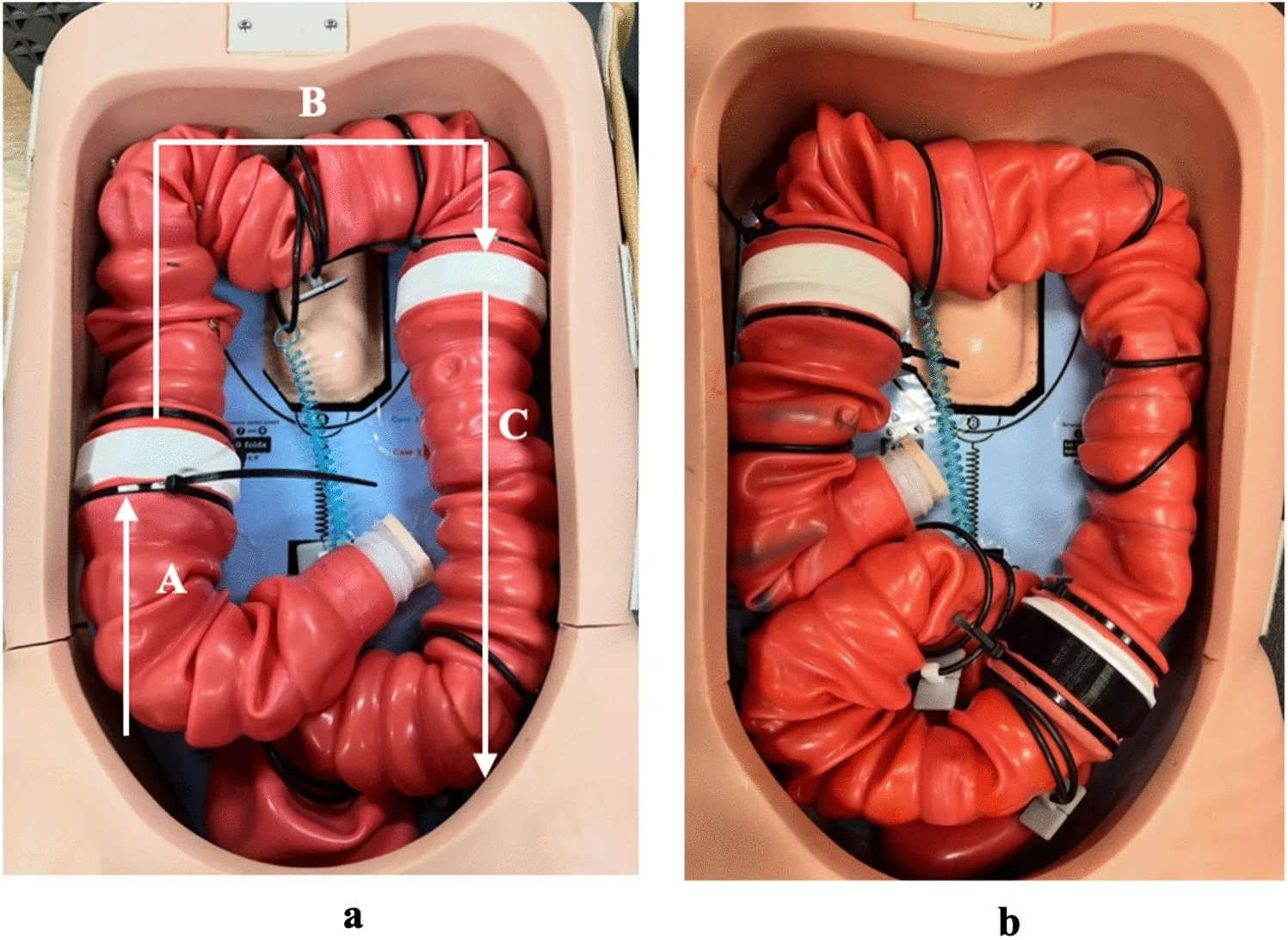
**a** The easy case with no loops with sections A, B and C in the Modified Kyoto Kagaku Simulator and **b** The moderate case with an alpha loop

**Fig. 5:**
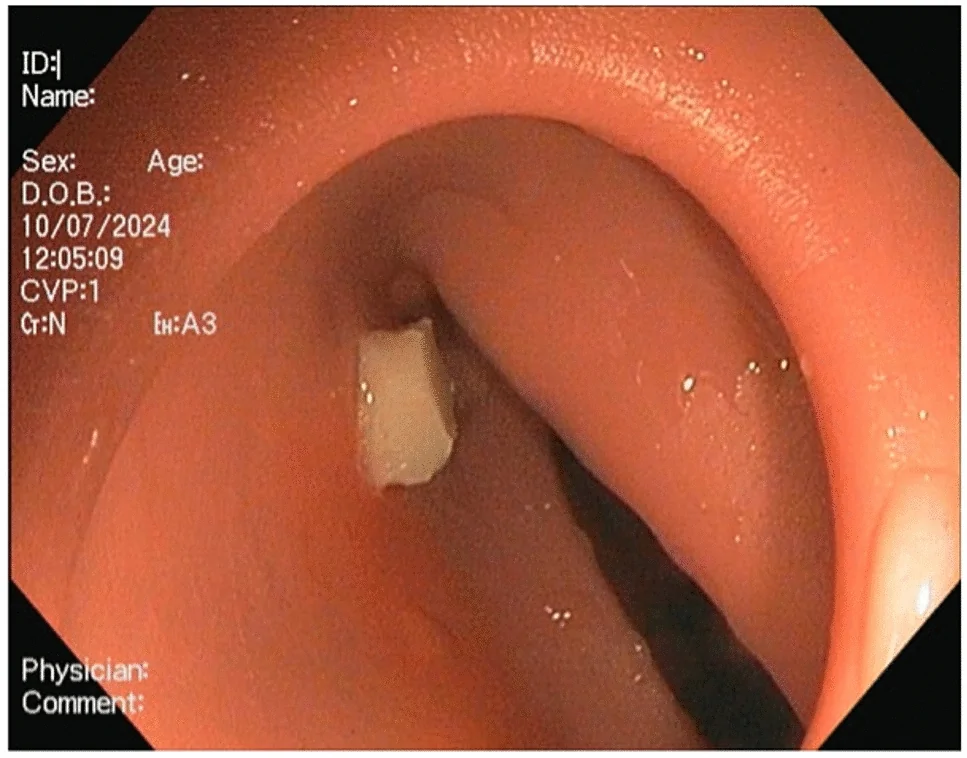
A silicone replica of a polyp inside the modified Kyoto Kagaku Simulator

A Kyoto Kagaku Physical Colonoscopy Simulator [48], was modified to include 15 artificial silicone polyps (1–5 mm) for this study (Fig. 4a). The simulator consists of a flexible rubber colon tube inside a life-size manikin and can be configured into six difficulty levels; the easy (case 1) and moderate looped (case 2) configurations were used (Fig. 4a, b), polyps were created by mixing equal portions of silicone components, cutting them into 1–5 mm circular shapes, and verifying their size, see Fig. 5. To embed the polyps, the colon was divided into three segments (A, B, C) at two randomly selected points, see Fig. 4a. Internal folds and bends were marked in the moderate case configuration, and five polyps were attached within each segment using Gorilla Glue.

**Fig. 6:**
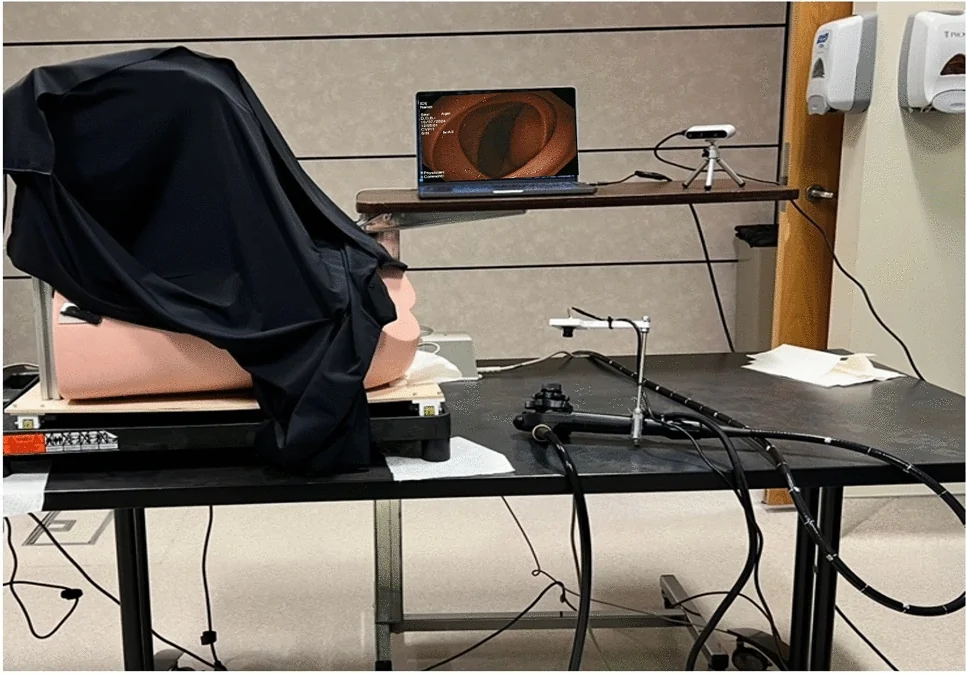
A setup of the equipment used in the study which includes the manikin, computer, endoscope and cameras

Additional equipment included a standard Olympus Endoscope Evis Exera, with the camera output displayed on a 14‑inch MacBook Pro positioned on an adjustable table to align with participants’ eye level. Tobii Pro 3 eye trackers (100 Hz sampling rate) were used to record gaze behavior throughout the study. The experimental setup is illustrated in Fig. 6.

#### Participants

Participants were recruited through a program coordinator at Hershey Medical Center (HMC), who distributed a recruitment email to general surgery residents. Individuals were screened for basic endoscope-handling knowledge before enrollment. The final sample included 5 experts and 12 residents. Experts were defined as surgeons with over 250 colonoscopies, consistent with evidence that competence typically requires this level of experience [49]. Residents were categorized as intermediates (11–100 colonoscopies, *n* = 5) or novices (0–10 colonoscopies, *n* = 7), based on prior work [50]. Participant demographics are shown in Table 2.

**Table 2:** Demographics of participants

	Experts	Residents
Total participants	5	12
Gender		
Male	4	10
Female	1	2
Ethnicity		
White	3	10
Asian	2	1
Hispanic/Latino	1	0
Black/African American	0	1
American Indian/Alaska Native	0	0
More than one race	0	0
Specialty		
General surgery	5	10
Emergency medicine	0	1
Anesthesia	0	1
Years of experience in specialty		
0	0	2
1–4	0	8
5–6	4	2
> 13	1	0
Previous colonoscopy experience		
# of colonoscopies performed		
0	0	4
1–10		3
20–50	0	3
70–80		2
250–275	2	0
> 400	1	0
1000 or higher	2	0
Previous training		
None	0	1
Simulator	5	11
Hands-on training	5	6
Fellowship	4	0

#### Procedure

This study represents a subset of a larger investigation; only the methods relevant to the present analysis are described here. At the beginning of the study, informed consent was obtained from all participants in accordance with institutional review board protocols. Each participant completed a brief survey on prior experience. Participants were then randomized to either case 1 (C1) or case 2 (C2). Group allocation included 2 experts, 3 intermediates, and 2 novices in C1, and 3 experts, 2 intermediates, and 5 novices in C2.

**Fig. 7:**
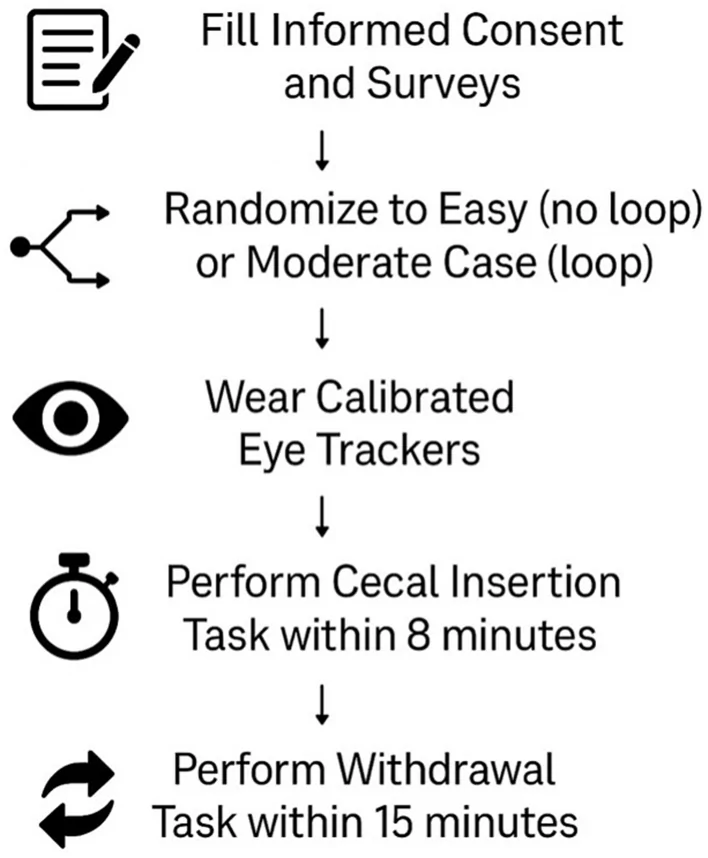
Steps of study procedure

Because this project was part of a larger study requiring eye-tracking, participants wore individually calibrated Tobii Pro 3 Glasses although eye-tracking data was not used in the present analysis. Participants were given up to 8 min to reach the cecum—based on prior work indicating experts typically require 5 min, with extra time allotted residents [51]. If they could not reach the cecum within the allotted time, the research team advanced the scope to the cecum. Participants then performed the withdrawal task, were shown an example of a polyp beforehand, and were instructed to keep the endoscope close to each polyp once detected. Steps of the procedure is shown in Fig. 7.

## Results

This section highlights our results in terms of the RQs. All statistics were analyzed with SPSS (v. 30.0) with a significance level of 0.05.

### RQ1: Does the time spent in each inspection technique during withdrawal relate to polyp detection rate (PDR), and can the proportion of inspection time across techniques predict PDR?

Our first research question was developed to investigate whether inspection time by technique relates to PDR, and whether the proportion of inspection time in each technique can predict PDR.

A Pearson product–moment correlation was conducted to examine the relationship between time spent in each inspection technique (Technique 1–3) and PDR. Assumptions were met: the relationship was linear only for Technique 3, both variables were normally distributed according to Shapiro–Wilk tests (*p* > 0.05), and no outliers were detected. Time spent inspecting in Technique 1 (lumen fully visible and endoscope roughly centered) showed a small, non-significant correlation with PDR, *r*(15) = 0.145, *p* = 0.580. Similarly, time in Technique 2 (lumen partially visible) showed a small, non-significant correlation, *r*(15) = 0.297, *p* = 0.247. Time spent inspecting in Technique 3 (lumen not visible; deeper, more obscured areas such as folds/bends) demonstrated a strong, positive correlation with PDR, *r*(15) = 0.50, *p* = 0.041.

**Fig. 8:**
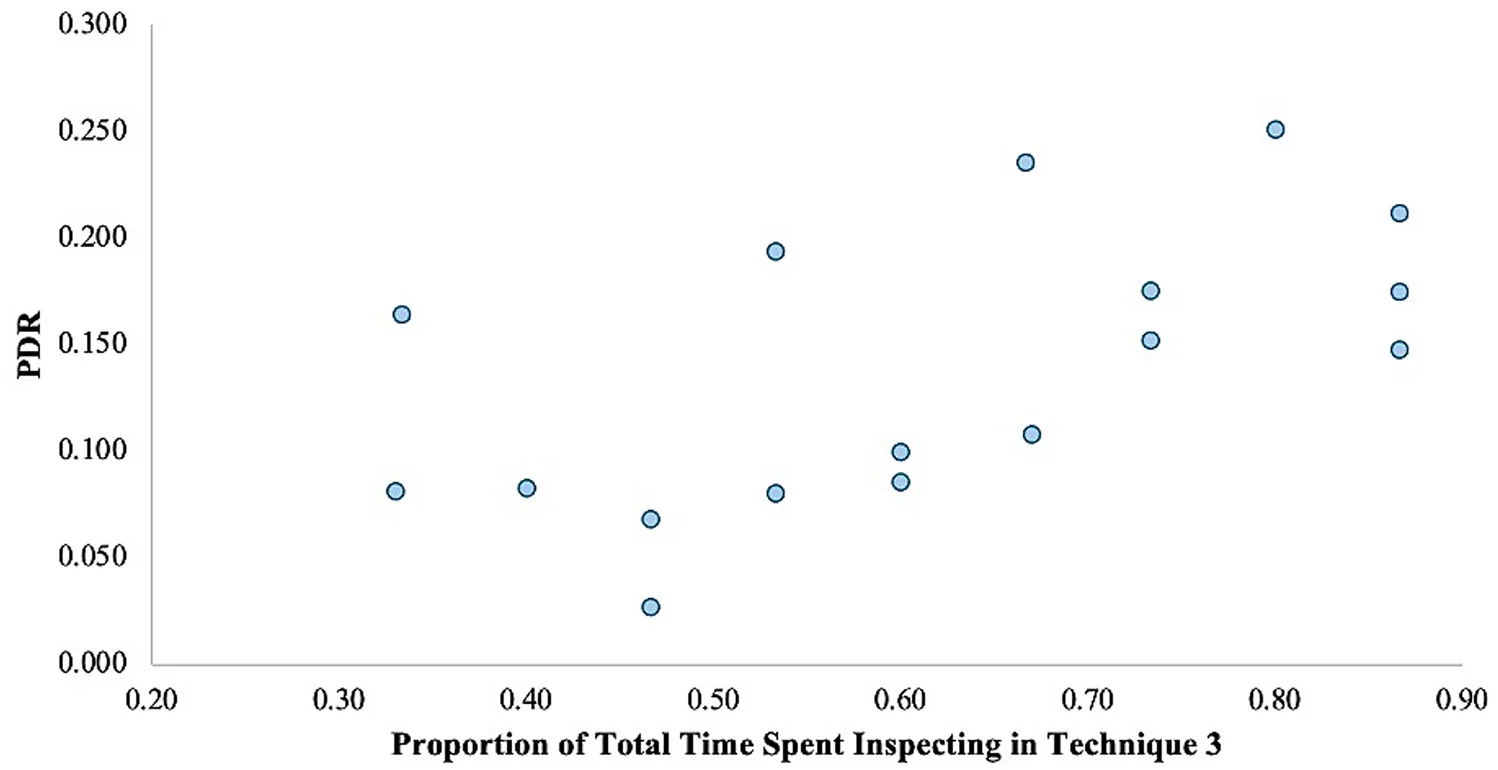
Scatterplot illustrating linear relationship between proportion of time spent inspecting in Technique 3 (lumen not visible) and PDR

A linear regression was then conducted to determine whether the proportion of time spent in each inspection technique predicted PDR. As no significant linear relationships were found for Technique 1 or 2, only Technique 3 was examined further. Visual inspection of scatterplots (Fig. 8) confirmed linearity, and assumptions of homoscedasticity, normality of residuals, and absence of outliers were met. The proportion of time spent inspecting in Technique 3 significantly predicted PDR, F(1, 15) = 7.834, *p* = 0.013, explaining 34.3% of the variance (adjusted *R*^2^ = 29.9%). Each additional second of proportional time spent in Technique 3 was associated with a 1.646 increase in PDR (95% CI [0.393, 2.9]).

### RQ2: Does PDR and inspection time across the three techniques differ between experts, intermediates, and novice residents during colonoscopy withdrawal?

Our second research question was developed to understand whether there are differences in PDR and inspection time in each technique between the different expertise levels.

Descriptive statistics of inspection time by technique and PDR is shown in Table 3. Since preliminary analysis using one-way ANOVAs revealed that condition (easy vs. moderate) did not significantly affect time spent in Technique 1–3 or PDR, the analyses were run without condition. A one-way ANOVA was run to examine differences in Technique 1 inspection time (lumen fully visible) across expertise levels. There were no outliers, assumptions of normality (Shapiro–Wilk, *p* > 0.05) and homogeneity of variances (Levene’s *p* = 0.094) were met. Time spent inspecting in Technique 1 was statistically significantly different for different levels of expertise, F(2, 14) = 3.865, *p* = 0.046, *η*^2^ = 0.356. Mean inspection time increased from novices (*M* = 27.1 s, SD = 8.19), to intermediates (*M* = 33.8 s, SD = 21.4), to experts (*M* = 51.3 s, SD = 15.32). Tukey tests showed experts spent significantly more time than novices (mean difference = 24.2 s, 95% CI [1.15, 47.32], *p* = 0.039); no other group differences were significant. A second one-way ANOVA assessed Technique 2 inspection time (lumen partially visible). No outliers were detected, and assumptions of normality (*p* > 0.05) and equal variances (Levene’s *p* = 0.874) were met. Mean Technique 2 inspection times increased from novices (*M* = 198.5 s, SD = 68.8), to experts (*M* = 251.2 s, SD = 81.9), to intermediates (*M* = 279.1 s, SD = 81.8), but the effect was not significant, F(2, 14) = 1.716, *p* = 0.213, *η*^2^ = 0.198.

**Table 3:** Descriptive statistics for PDR, and total time inspecting in technique 1, 2 and 2 across experts, intermediates and novice residents

Metric	Experts (*n* = 5)	Intermediate (*n* = 5)	Novices (*n* = 7)
PDR	0.76 ± 0.077	0.64 ± 0.209	0.49 ± 0.14
Time in Technique 1 (s)	51.32 ± 15.32	33.8 ± 21.4	27.1 ± 8.19
Time in Technique 2 (s)	251.2 ± 81.9	279.1 ± 81.8	198.5 ± 68.8
Time in Technique 3 (s)	83.0 ± 34.7	49.56 ± 40.0	26.5 ± 12.71

Given the significant correlation found between Technique 3 inspection time and PDR (RQ1), a one-way MANOVA was conducted to examine differences in Technique 3 inspection time (deeper, obscured areas) and PDR across expertise levels. Assumptions were satisfied: normality (Shapiro–Wilk, *p* > 0.05), no univariate or multivariate outliers (boxplots; Mahalanobis *p* > 0.001), linearity, no multicollinearity (*r* = 0.50, *p* = 0.041), and homogeneity of variance–covariance matrices (Box’s M = 0.256). The differences between the expertise levels on the combined dependent variables was statistically significant, F(4, 26) = 3.262, *p* = 0.027; Wilks’ *Λ* = 0.443; partial *η*^2^ = 0.334.

Follow-up ANOVAs showed significant group differences in both Technique 3 inspection time, F(2, 14) = 5.345, *p* = 0.019, partial *η*^2^ = 0.433, and PDR, F(2, 14) = 4.659, *p* = 0.028, partial *η*^2^ = 0.400. Post hoc Bonferroni tests indicated that experts had significantly higher PDR than novices (mean difference = 0.2657, SE = 0.088, *p* = 0.024, 95% CI [0.0264, 0.505]) but did not differ from intermediates (*p* = 0.705). Experts also spent significantly more time in Technique 3 than novices (mean difference = 56.48 s, SE = 17.3, *p* = 0.017, 95% CI [9.52, 103.4]), with no significant difference between experts and intermediates (*p* = 0.458).

### RQ3: Do experts, intermediates, and novices differ in the time spent inspecting in each technique before and after polyp detection during withdrawal?

Our third research question was developed to understand whether there are differences between experts compared to residents in inspection time in the different inspection techniques, before and after a polyp was detected.

Three linear mixed-effects models were conducted to examine differences in time spent in each of the three inspection techniques within a ± 6-s window before and after polyp detection among experts, intermediates, and novice residents (see Sect. "Outcome measures"). Fixed effects included expertise level, detection window (pre vs. post), and their interaction. Intraclass correlation coefficients were sufficient with 31.9%, 33.1% and 32.8% across all three inspection techniques—Technique 1 (lumen fully visible), Technique 2 (wall view with partially visible lumen), and Technique 3 (deeper, obscured regions such as folds) respectively—supporting the inclusion of participant ID as a random intercept to account for multiple polyp detection events per participant. Assumptions of linearity and normality of residuals were met. Estimated marginal means were reported, and Bonferroni adjustments were applied for post hoc comparisons.

For time spent in Technique 1 within the ± 6-s window surrounding polyp detection, the linear mixed-effects model revealed a significant main effect of expertise, *F*(2, 202) = 7.22, *p* < 0.001. Neither the main effect of detection window, *F*(1, 202) = 0.26, *p* = 0.610, nor the expertise × window interaction, *F*(2, 202) = 2.31, *p* = 0.102, was significant. Post hoc comparisons indicated that overall before or after polyp detection, novices (*M* = 1.290 s, SE = 0.998 s) spent significantly more time in Technique 1 than experts (*M* = 0.618 s, SE = 0.998 s, *p* = 0.009), whereas no significant difference was observed between experts and intermediates (*p* = 1.00). Parameter estimates and 95% confidence intervals are reported in Table 4.

**Table 4:** Fixed effects, random effects, and model fit results from the linear mixed-effects model examining time spent in inspection Technique 1 (lumen fully visible) within a ± 6-s window before and after polyp detection

	Fixed effects			
Group	B	SE	95% CI	*p*
Novices	1.133	0.316	[0.510, 1.755]	< 0.001
Intermediates	0.195	0.343	[−0.481, 0.870]	0.571

	Random effects	
Participant (intercept)	Variance	SD
	0.688	0.829

	Model fit	
R^2^	Marginal	Conditional
	0.033	0.361

Reference category for expertise was experts. Random effects represent participant-level variability. Marginal *R*^2^ reflects variance explained by fixed effects only, whereas conditional *R*^2^ reflects variance explained by both fixed and random effects

For time spent in Technique 2, the linear mixed-effects model only revealed a significant main effect of expertise, *F*(2, 202) = 3.637, *p* = 0.028, with marginal and conditional R squared of 0.026 and 0.357, respectively. Post hoc comparisons indicated that this effect was driven by differences between novices (*M* = 4.284 s, SE = 1.142 s) and intermediates (*M* = 5.01 s, SE = 1.147 s, *p* = 0.027), whereas expert time (*M* = 4.47 s, SE = 1.142 s) did not differ significantly from intermediates (*p* = 0.150) or novices (*p* = 1.00).

For time spent in Technique 3, the linear mixed-effects model revealed a significant main effect of expertise, *F*(2, 202) = 4.361, *p* = 0.014. Neither the main effect of detection window, *F*(1, 202) = 0.03, *p* = 0.863, nor the expertise × window interaction, *F*(2, 202) = 0.989, *p* = 0.374, was significant. Post hoc comparisons indicated that overall before or after polyp detection, experts (*M* = 0.937 s, SE = 0.840 s) spent significantly more time in Technique 3 than novices (*M* = 0.409 s, SE = 0.840 s, *p* = 0.016) but not compared to intermediate residents (*M* = 0.511 s, SE = 0.844 s, *p* = 0.113). Parameter estimates and 95% confidence intervals are reported in Table 5.

**Table 5:** Fixed effects, random effects, and model fit results from the linear mixed-effects model examining time spent in inspection Technique 3 (deeper, obscured regions like folds) within a ± 6-s window before and after polyp detection

	Fixed effects			
Group	B	SE	95% CI	*p*
Novices	−0.793	0.266	[−1.317, −0.269]	0.003
Intermediates	−0.564	0.288	[−1.132,0.005]	0.052

	Random effects	
Participant (intercept)	Variance	SD
	0.971	0.985

	Model fit	
R^2^	Marginal	Conditional
	0.058	0.377

Reference category for expertise was experts. Random effects represent participant-level variability. Marginal *R*^2^ reflects variance explained by fixed effects only, whereas conditional *R*^2^ reflects variance explained by both fixed and random effects

## Discussion

The goal of this exploratory feasibility study was to improve colonoscopy training by providing automatic object assessment of inspection performance and identifying targeted feedback for physical SBT which may help improve mucosal inspection quality and ADR. This was achieved by training and validating a ViT-based CVCM model to automatically recognize the three key inspection techniques during colonoscopy withdrawal. The ViT-based CVCM demonstrated strong performance on simulator withdrawal data, achieving high accuracy and precision, recall, and F1-scores across all inspection techniques. Overall, these findings highlight the ViT model’s promise for accurately recognizing key inspection techniques during colonoscopy withdrawal and its potential as a first step in advancing automated assessment of inspection performance using inspection time in each technique during physical SBT.

For the **first research question**, our hypothesis was that longer inspection time in each technique would be correlated with higher PDR and may predict PDR. Our results partially support this since there was only a strong, positive significant correlation (*r* = 0.51) between time spent inspecting in deeper, more obscured areas of the colon (technique 3) and PDR, and that time spent inspecting in technique 3 can predict PDR. This may indicate that more thorough inspection in the deeper and obscured areas in the colon, which may also include behind mucosal folds or bends, may have potentially contributed to the detection of polyps in these difficult-to-visualize regions. This further highlights the importance of this inspection technique since research has shown that interval CRC can result from polyps hidden behind folds or valves that are missed during inspection [33]. In addition, research has shown that inspection behind folds lowers miss rate of polyps [28] and difficulty in visualizing behind folds and flexures can have a negative impact on ADR [34].

Results showed a small, non-significant correlation between time spent inspecting with clear lumen view (technique 1) and PDR. Two potential explanations could be related to the small sample size of participants or that maintaining a full, centered lumen view may not directly contribute to higher PDR. However, this technique may still be valuable, as it can provide an optimal reference point from which to systematically inspect the surrounding mucosa and deeper areas behind folds or bends. Thus, while technique 1 behavior alone may not predict PDR, it may facilitate more effective inspection overall. This is because previous work emphasized that the endoscope should be actively redirected from the lumen center toward the lateral walls for colonic inspection [32]. Similarly, a small, non-significant correlation was found between time spent in Technique 2 (lumen partially visible, inspecting lateral walls) and PDR. In addition to sample size limitations, spending more time inspecting the sides of the colon may not guarantee that all regions were thoroughly examined. Future work should evaluate not only inspection time but also the completeness and quality of mucosal inspection. This is because the quality of the inspection may be more important than inspection time alone for detecting polyps [52].

For the **second research question**, our hypothesis was that inspection time across techniques would differ by expertise level and that experts would achieve higher PDR than residents. Our results mainly support this since, first, experts spent more time inspecting with a clear lumen view and a centered endoscope (Technique 1) compared to novices only, with a large eta-squared effect size (*η*^2^ > 0.1379) [53]. This suggests that experts and intermediates may have inspected more of the mucosa while maintaining optimal visualization and systematically extending inspection to surrounding walls and deeper areas. These findings align with prior work showing that experts inspect mucosa while maintaining a clear lumen view lumen [22], and that frequent re-centering of the endoscope is an expert strategy for thorough mucosal evaluation [23]. In addition, achieving a clear lumen view is recognized as a core technical skill inspection [30], and proper positioning of the colonoscope tip is essential for optimal visualization of the colonic lumen during withdrawal lumen [31].

Secondly, results also indicated that experts spent more time inspecting deeper, more difficult-to-visualize and obscured areas of the colon that may include folds/bends (Technique 3), and achieved higher PDR compared to novices only, with a large effect (*η*^2^ > 0.1379) These findings suggest that experts and intermediates may have potentially conducted more thorough mucosal inspection, particularly in regions where polyps are often missed, enabling greater detection. Prior work supports this, showing that expert surgeons typically inspect most of the simulated colonic mucosa [29]. One possible explanation is the use of advanced techniques, such as inspection behind internal areas [26, 27], “pushing down folds” [29], and fold inspection [23], which are associated with higher ADR. Experts and intermediates may have been more familiar with these techniques, whereas novices may not have. Further research is needed to confirm whether these other techniques were used.

Results also indicated no significant differences among experts, intermediates, and novices in inspection time when examining the lateral sides of the colon wall with the lumen partially visible (Technique 2), with a large effect (*η*^2^ > 0.1379). A possible explanation is that experts devoted more time to clear lumen views (Technique 1) and deeper, obscured areas (Technique 3), whereas novices spent most of their time in Technique 2. This distribution may account for the lack of group differences in total time spent in Technique 2. Prior work has shown that experts and novices exhibit comparable left–right movement patterns during colonoscopy withdrawal on VR simulators, possibly suggesting similar attempts to inspect lateral walls [29]. An important question that will be investigated in future work and will be used to further validate these findings is whether novices, despite spending more time in this technique or the other techniques, were conducting thorough inspection of all lateral surfaces or merely passing through without complete and effective mucosal inspection.

For the **third research question**, our hypothesis was that expertise level would influence inspection time within a time window surrounding polyp detection, particularly in deeper, more obscured areas. Our results partially support this since no differences were observed between experts and residents in lateral wall inspection time (lumen partially visible), with a small marginal *R*^2^ effect size (< 0.04) [54]. However, overall across both pre and post polyp detection windows, only novices spent more time maintaining a clear lumen view (marginal *R*^2^ = 0.033), whereas experts devoted more time to deeper, obscured region (marginal *R*^2^ = 0.058), both reflecting small effect sizes. Given the relatively small sample size, these results may have been underpowered to detect subtle behavioral differences between expertise groups, potentially contributing to the small observed effect sizes and non-significant findings. However, this may suggest that experts and intermediates may have briefly redirected their attention toward inspecting difficult-to-visualize obscured regions, possibly to ensure no additional polyps were hidden nearby. To our knowledge, prior studies have not examined inspection behavior specifically before and after polyp detection. These findings highlight the potential value of feedback during SBT to encourage novices to inspect deeper, obscured areas surrounding polyp detection. Although the observed differences were brief (≤ 1 s), even short inspection periods may be meaningful given the rapid processing of visual motion by humans in under 150 ms [55]. However, further research with larger sample size, extended analysis windows and follow up interviews is needed to validate these results.

Although further validation with larger samples is needed, this study demonstrates the potential of the ViT-based CVCM to automatically and accurately recognize key inspection techniques during withdrawal and objectively differentiate performance based on inspection time, particularly in clear lumen views and difficult-to-visualize obscured regions. With continued development and further validation through an analysis of mucosal coverage, this ML approach could provide objective metrics for assessing mucosal inspection quality in physical SBT. The findings also suggest real-time feedback strategies for simulators, such as prompting re-centering for clear lumen views, highlighting uninspected or obscured regions behind folds, and encouraging inspection in deeper and more difficult-to-visualize regions before and after polyp detection. This feedback may enhance inspection performance and reduce missed polyps, since feedback can improve ADR [38].

## Limitations

While this study provides promising insights for the use of ML in automatically and objectively assessing inspection performance and identifying feedback for physical SBT, several limitations must be addressed to enhance generalizability and guide future work. First, it was conducted at a single medical institution with limited participant availability. A larger and more diverse sample will be needed to improve generalizability and strengthen the validity of the findings. In addition, given the limited sample size of 17, the study may have been underpowered to detect small-to-moderate differences between groups. Therefore, some small or non-significant findings should be interpreted with caution, as they may reflect limited statistical power rather than absence of a true effect. Future studies with larger samples are needed to confirm these findings. Second, although the colon model configuration was standardized, slight variations in the 3D connectors or participant scope movements may have shifted polyp positions, potentially influencing PDR. Future work will explore more precise methods for placing polyp replicas to ensure consistent positioning across participants. Lastly, participants were restricted from using insufflation and suction valves. While these functions may be more advanced for novices, they could have improved visualization for more experienced participants and potentially increased PDR. Future research should consider integrating these features to better reflect real-world practice.

## Conclusion

This exploratory feasibility study demonstrated the ability of a ViT-based CVCM to automatically and accurately recognize key inspection techniques (clear lumen view, lateral wall view and deeper, more obscured inspection) and assess colonoscopy inspection performance using time spent in each inspection technique and PDR. The ViT CVCM achieved strong accuracy and performance in simulator data. Findings showed that increased inspection time in deeper, obscured regions was the only behavior significantly associated with higher PDR, and that experts devoted more time in clear lumen views, and in difficult-to-visualize regions both overall and before or after polyp detection compared to novices. These results highlight the value of the ViT CVCM in objectively distinguishing expertise levels and supporting the integration of targeted feedback—such as re-centering or highlighting uninspected obscured regions—into simulation-based training. Future work should validate these findings with larger samples and evaluate the impact of feedback strategies on inspection quality and PDR.

## References

[1] Siegel RL, Giaquinto AN, Jemal A (2024) Cancer statistics, 2024. CA Cancer J Clin 74(1):12–49. 10.3322/caac.21820 38230766

[2] Benson AB, Venook AP, Al-Hawary MM, Cederquist L, Chen Y-J, Ciombor KK, Cohen S, Cooper HS, Deming D, Engstrom PF (2018) NCCN guidelines insights: colon cancer, version 2.2018. J Natl Compr Canc Netw 16(4):359–369 10.6004/jnccn.2018.0021 PMC10184502 29632055

[3] Baxter NN, Warren JL, Barrett MJ, Stukel TA, Doria-Rose VP (2012) Association between colonoscopy and colorectal cancer mortality in a US cohort according to site of cancer and colonoscopist specialty. J Clin Oncol 30(21):2664–2669 10.1200/JCO.2011.40.4772 PMC3413278 22689809

[4] Araghi M et al (2019) Global trends in colorectal cancer mortality: projections to the year 2035. Int J Cancer 144(12):2992–3000. 10.1002/ijc.32055 30536395

[5] Ahmed M (2019) Colon cancer: a clinician’s perspective in 2019. Gastroenterol Res 13(1):1 10.14740/gr1239 PMC7011914 32095167

[6] Wolf AMD et al (2018) Colorectal cancer screening for average‐risk adults: 2018 guideline update from the American Cancer Society. CA Cancer J Clin 68(4):250–281. 10.3322/caac.21457 29846947

[7] Patel KS et al (2022) Current trends and predictors of case outcomes for malpractice in colonoscopy in the United States. J Clin Gastroenterol 56(1):49–54. 10.1097/MCG.0000000000001471 33337638

[8] Sullivan BA, Noujaim M, Roper J (2022) Cause, epidemiology, and histology of polyps and pathways to colorectal cancer. Gastrointest Endosc Clin N Am 32(2):177–194 10.1016/j.giec.2021.12.001 PMC9924026 35361330

[9] Lee SH, Chung IK, Kim SJ, Kim JO, Ko BM, Hwangbo Y, Kim WH, Park DH, Lee SK, Park CH, Baek IH, Park DI, Park SJ, Ji JS, Jang BI, Jeen YT, Shin JE, Byeon JS, Eun CS, Han DS (2008) An adequate level of training for technical competence in screening and diagnostic colonoscopy: a prospective multicenter evaluation of the learning curve. Gastrointest Endosc 67(4):683–689 10.1016/j.gie.2007.10.018 18279862

[10] Winawer SJ, Zauber AG, Ho MN, O’Brien MJ, Gottlieb LS, Sternberg SS, Waye JD, Schapiro M, Bond JH, Panish JF, Ackroyd F, Shike M, Kurtz RC, Hornsby-Lewis L, Gerdes H, Stewart ET, National Polyp Study Workgroup (1993) Prevention of colorectal cancer by colonoscopic polypectomy. New Engl J Med 329(27):1977–1981 10.1056/NEJM199312303292701 8247072

[11] Hsu W, Chiu H (2023) Optimization of colonoscopy quality: comprehensive review of the literature and future perspectives. Dig Endosc 35(7):822–834. 10.1111/den.14627 37381701

[12] Anderson R, Burr NE, Valori R (2020) Causes of post-colonoscopy colorectal cancers based on World Endoscopy Organization system of analysis. Gastroenterology 158(5):1287-1299.e2. 10.1053/j.gastro.2019.12.031 31926170

[13] Pore A et al (2022) Colonoscopy navigation using end-to-end deep visuomotor control: a user study. In: 2022 IEEE/RSJ International Conference on Intelligent Robots and Systems (IROS), IEEE, pp 9582–9588. 10.1109/IROS47612.2022.9981480

[14] Corley DA et al (2014) Adenoma detection rate and risk of colorectal cancer and death. N Engl J Med 370(14):1298–1306. 10.1056/NEJMoa1309086 PMC4036494 24693890

[15] Rex DK et al (2002) Quality in the technical performance of colonoscopy and the continuous quality improvement process for colonoscopy: recommendations of the U.S. Multi-Society Task Force on Colorectal Cancer. Am J Gastroenterol 97(6):1296–1308. 10.1111/j.1572-0241.2002.05812.x 12094842

[16] Sedlack RE (2013) The state of simulation in endoscopy education: continuing to advance toward our goals. Gastroenterology 144(1):9–12. 10.1053/j.gastro.2012.11.007 23149221

[17] Accreditation Council for Graduate Medical Education. Defined category minimum numbers for general surgery residents and credit role. https://www.acgme.org/globalassets/DefinedCategoryMinimumNumbersforGeneralSurgeryResidentsandCreditRole.pdf. Accessed 15 Nov 2021

[18] van Rijn JC, Reitsma JB, Stoker J, Bossuyt PM, van Deventer SJ, Dekker E (2006) Polyp miss rate determined by tandem colonoscopy: a systematic review. Am J Gastroenterol 101(2):343–350 10.1111/j.1572-0241.2006.00390.x 16454841

[19] Dik VK, Moons LM, Siersema PD (2014) Endoscopic innovations to increase the adenoma detection rate during colonoscopy. World J Gastroenterol 20(9):2200–2211. 10.3748/wjg.v20.i9.2200 PMC3942825 24605019

[20] Munroe CA et al (2012) A tandem colonoscopy study of adenoma miss rates during endoscopic training: a venture into uncharted territory. Gastrointest Endosc 75(3):561–567. 10.1016/j.gie.2011.11.037 22341103

[21] Postgate A, Tekkis P, Fitzpatrick A, Bassett P, Fraser C (2008) The impact of experience on polyp detection and sizing accuracy at capsule endoscopy: implications for training from an animal model study. Endoscopy 40(06):496–501. 10.1055/s-2007-995590 18556804

[22] Phitayakorn R, Marks JM, Reynolds HL, Delaney CP (2009) Expert benchmark for the GI Mentor IITM. Surg Endosc 23(3):611–614. 10.1007/s00464-008-0166-8 18813977

[23] Elsaadany IK, Motamedi S, Moore JZ, Miller SR (2025) Polyp detection in colonoscopy training: a qualitative analysis of resident challenges and expert strategies. Proc Hum Factors Ergon Soc Annu Meet. 10.1177/10711813251374166 PMC13007603 41878495

[24] Liu X, Tavanapong W, Wong J, Oh J, de Groen PC (2010) Automated measurement of quality of mucosa inspection for colonoscopy. Procedia Comput Sci 1(1):951–960. 10.1016/j.procs.2010.04.105

[25] Liu D, Cao Y, Tavanapong W, Wong J, Oh J, de Groen PC (2007) Quadrant coverage histogram: a new method for measuring quality of colonoscopic procedures. In: 2007 29th Annual International Conference of the IEEE Engineering in Medicine and Biology Society, IEEE, pp. 3470–3473. 10.1109/IEMBS.2007.4353078 18002744

[26] Barclay RL, Vicari JJ, Greenlaw RL (2008) Effect of a time-dependent colonoscopic withdrawal protocol on adenoma detection during screening colonoscopy. Clin Gastroenterol Hepatol 6(10):1091–1098. 10.1016/j.cgh.2008.04.018 18639495

[27] Bourke MJ (2009) Making every colonoscopy count: Ensuring quality in endoscopy. J Gastroenterol Hepatol. 10.1111/j.1440-1746.2009.06070.x 19799697

[28] Lee RH et al (2011) Quality of colonoscopy withdrawal technique and variability in adenoma detection rates (with videos). Gastrointest Endosc 74(1):128–134. 10.1016/j.gie.2011.03.003 21531410

[29] Zupanc CM et al (2017) Assessing colonoscopic inspection skill using a virtual withdrawal simulation: a preliminary validation of performance metrics. BMC Med Educ 17(1):118. 10.1186/s12909-017-0948-6 PMC5508767 28701200

[30] Walsh CM et al (2021) Colonoscopy core curriculum. Gastrointest Endosc 93(2):297–304. 10.1016/j.gie.2020.06.054 32854938

[31] Pullens H et al (2015) Colonoscopy with robotic steering and automated lumen centralization: a feasibility study in a colon model. Endoscopy 48(03):286–290. 10.1055/s-0034-1392550 26126158

[32] de Groen PC (2020) Using artificial intelligence to improve adequacy of inspection in gastrointestinal endoscopy. Tech Innov Gastrointest Endosc 22(2):71–79. 10.1016/j.tgie.2019.150640

[33] Rex DK (2006) Maximizing detection of adenomas and cancers during colonoscopy. Am J Gastroenterol 101(12):2866–2877. 10.1111/j.1572-0241.2006.00905.x 17227527

[34] Rees CJ, Rajasekhar PT, Rutter MD, Dekker E (2014) Quality in colonoscopy: European perspectives and practice. Expert Rev Gastroenterol Hepatol 8(1):29–47. 10.1586/17474124.2014.858599 24410471

[35] Khan R, Plahouras J, Johnston BC, Scaffidi MA, Grover SC, Walsh CM (2018) Virtual reality simulation training for health professions trainees in gastrointestinal endoscopy. Cochrane Database Syst Rev. 10.1002/14651858.CD008237.pub3 PMC6513657 30117156

[36] Kaltenbach T, Krop L, Nguyen-Vu T, Soetikno R (2024) Improving adenoma detection and resection: the role of tools, techniques and simulation-based mastery learning. Tech Innov Gastrointest Endosc 26(2):167–176. 10.1016/j.tige.2024.03.004

[37] Goodman AJ, Melson J, Aslanian HR, Bhutani MS, Krishnan K, Lichtenstein DR, Navaneethan U, Pannala R, Parsi MA, Schulman AR, Sethi A, Sullivan SA, Thosani N, Trikudanathan G, Trindade AJ, Watson RR, Maple JT, ASGE Technology Committee (2019) Endoscopic simulators. Gastrointest Endosc 90(1):1–12 10.1016/j.gie.2018.10.037 31122746

[38] Scalvini D et al (2025) Strategies to enhance the adenoma detection rate (ADR) and the serrated polyp detection rate (SPDR) in colonoscopy: a comprehensive review. Gastroenterol Insights 16(1):9. 10.3390/gastroent16010009

[39] Van der Meijden OA, Schijven MP (2009) The value of haptic feedback in conventional and robot-assisted minimal invasive surgery and virtual reality training: a current review. Surg Endosc 23:1180–1190 10.1007/s00464-008-0298-x PMC2686803 19118414

[40] Finocchiaro M et al (2023) Physical simulator for colonoscopy: a modular design approach and validation. IEEE Access 11:36945–36960. 10.1109/ACCESS.2023.3266087

[41] Lavanchy JL et al (2021) Automation of surgical skill assessment using a three-stage machine learning algorithm. Sci Rep 11(1):5197. 10.1038/s41598-021-84295-6 PMC7933408 33664317

[42] Chen G et al (2025) Predicting bone metastasis risk of colorectal tumors using radiomics and deep learning ViT model. J Bone Oncol 51:100659. 10.1016/j.jbo.2024.100659 PMC11787686 39902382

[43] Altman DG (1990) Practical statistics for medical research. Chapman and Hall/CRC. 10.1201/9780429258589

[44] Dwyer B, Nelson J, Hansen T, et al (2026) Roboflow (Version 1.0) [Software]. Available from https://roboflow.com. Computer Vision

[45] Raschka S (2018) Model evaluation, model selection, and algorithm selection in machine learning. arXiv preprint arXiv:1811.12808

[46] Shorten C, Khoshgoftaar TM (2019) A survey on image data augmentation for deep learning. J Big Data 6(1):60. 10.1186/s40537-019-0197-0 PMC8287113 34306963

[47] Schaye V et al (2022) Development and validation of a machine learning model for automated assessment of resident clinical reasoning documentation. J Gen Intern Med 37(9):2230–2238. 10.1007/s11606-022-07526-0 PMC9296753 35710676

[48] Kyoto Kagaku Co., Ltd (2024) Colonoscopy training model M40: Instruction manual [PDF]. https://www.kyotokagaku.com/products_data/m40_manual_en.pdf

[49] Sedlack RE et al (2016) Assessment of competency in endoscopy: establishing and validating generalizable competency benchmarks for colonoscopy. Gastrointest Endosc 83(3):516-523.e1. 10.1016/j.gie.2015.04.041 26077455

[50] Mahmood T, Darzi A (2003) A study to validate the colonoscopy simulator. Surg Endosc 17(10):1583–1589. 10.1007/s00464-002-9222-y 12915972

[51] Plooy AM, Hill A, Horswill MS, Cresp AS, Watson MO, Ooi SY, Riek S, Wallis GM, Burgess-Limerick R, Hewett DG (2012) Construct validation of a physical model colonoscopy simulator. Gastrointest Endosc 76(1):144–150 10.1016/j.gie.2012.03.246 22726473

[52] Chaptini L, Laine L (2015) Can I improve my adenoma detection rate? J Clin Gastroenterol 49(4):270–281. 10.1097/MCG.0000000000000293 25647725

[53] Richardson JTE (2011) Eta squared and partial eta squared as measures of effect size in educational research. Educ Res Rev 6(2):135–147. 10.1016/j.edurev.2010.12.001

[54] Sullivan GM, Feinn R (2012) Using effect size—or why the P value is not enough. J Grad Med Educ 4(3):279–282. 10.4300/JGME-D-12-00156.1 PMC3444174 23997866

[55] Thorpe S, Fize D, Marlot C (1996) Speed of processing in the human visual system. Nature 381(6582):520–522. 10.1038/381520a0 8632824

